# The Burning-wells of Kenawha

**Published:** 1844-04

**Authors:** 


### THE BURNING-WELLS OF KENAWHA.

The following account of a most curious phenomenon, although not strictly professional, will be read, we are quite sure, with interest. We are indebted for it to Mr. James Putney, of Virginia, lately a pupil in the Medical Institute of Louisville. Y.

The Kenawha salt-works of Virginia are confined to the banks of the river of that name, and extend eight or ten miles up and down the stream. These works have been, and are yet, the source of most of the domestic salt consumed in the west, and have been in active operation for more than thirty years. The salt-water by which this supply is afforded is obtained by sinking a well on the brink of the river, at the point of low-water-mark, perforating the solid rocks by a hole not more than two or three inches in diameter; and it is a singular fact, that the salt-water is not to be obtained at any other point—not half a mile from the brink of the river. By a very tedious process, the rock has been penetrated to the depth of from six to twelve hundred feet, and in a few instances to even a greater depth. This operation was formerly accomplished by hand, or by horse-power, but is now done by means of steam. In boring to this immense depth, nothing is met with but the solid sand-stone rock, with the exception of two or three strata of bituminous coal. These strata are from a few inches to six feet in thickness, and are found only in the first five hundred feet. Fissures are sometimes met with in the rock which are a few inches in depth, and which contain water, or gas, sometimes both. As soon as the auger reaches these cavities the gas issues with great force, and is quickly exhausted. When a good fountain of salt-water is attained by the auger, it rises nearly to the level of the river, and is then pumped out, and forced into the reservoir for use.

To all who are acquainted with the history of these works it is known, that there exists in this county a remarkable phenomenon, which has received the common appellation of the *Burning Springs.* These are nothing more than excavations in the earth, capable of containing sixty or eighty gallons, and which, in a wet season, are filled with water. The gas rising through the water gives it the appearance of a great boiling caldron, which, if a torch be applied, instantly takes fire, and burns with a beautiful lambent flame. This has excited great fear and astonishment among the ignorant and superstitious, from the circumstance of the water's boiling, and apparently burning on its surface, and yet remaining cold. But a more recent exhibition of this phenomenon is to be seen in the *Gas-wells*, a short description of which I will now proceed to give.

A gentleman in boring his well, after having penetrated about one thousand feet, was surprised at an immense rush of gas, the carburetted hydrogen, mixed with strong salt-water. The gas was expelled with such force that it threw the water at least a hundred feet into the air, forming a most beautiful *jet d'eau*, and covering the earth with its white briny foam. The gas and water thus continued to be thrown out for many weeks, when the owner of the well began to think of turning it to some useful purpose. He proceeded to adjust suitable pipes to the well, and conveyed the gas to the reservoir on the top of the bank, the gas forcing the water before it with great power. By an apparatus fixed for the purpose, the water was separated from the gas, and both were conveyed to the furnace—the water into the pans, and the gas to the fire, to be applied to the bottoms of the pans. The whole furnace was thus filled with flame by which it was found that a saving of two-thirds of the fuel was effected. It was estimated that the quantity of gas emitted in twenty-four hours was sufficient to evaporate sixteen hundred gallons of water. The pipes in which it was conveyed to the furnace were five inches in diameter, and yet such was the force with which it was expelled, that a man was not capable of holding his hand upon the mouth of one of the pipes. A constant stream continues to issue from the well, its velocity, every few seconds, being much accelerated, in which it very much resembles the escape of steam from the engine of one of our largest steamboats, not, however, with the same regularity, nor with such distinct intermissions. The process of manufacturing salt with this gas is exceedingly interesting. The gas first expels the salt-water from the bottom of the well, and forces it into the reservoir. Then being ignited beneath the furnace, it converts the salt-water into brine. The brine is next conveyed into a wooden vat, and is then converted into salt, by the agency of the same steam which was generated in converting the water into brine.

The eruption of the gas at this point occurred about two years since, and it has continued to be emitted with the same copiousness ever since. A few months after its first expulsion another well was bored at a distance from it of about a hundred yards with similar results, the quantity of gas discharged being fully equal to that of the first. Since that time six other wells have afforded the same phenomenon, and one of these in particular deserves notice on account of the vast quantity of the gas and water thrown out. This is about a mile distant from the first. This well, it is computed, will supply salt-water in sufficient quantities to make a thousand bushels of salt in twenty-four hours, and enough gas to convert forty or fity thousand gallons of water into steam. In some of these wells the salt-water is, unfortunately, not very strong, and it is found a difficult matter to bore them to a greater depth.

An occurrence lately took place in one of these wells worthy of notice. After having emitted the gas for some time, the escape suddenly ceased, and in the belief that some obstruction was offered to its egress, the workmen were ordered to sink the augers and remove the obstacle. Suddenly a trembling of the earth was observed for many yards around the well, followed by a violent explosion, and a forcible expulsion of the augers, and a flame of fire—which presented a beautiful, and even sublime spectacle. The gas continued to burn until it was extinguished by the violence of the winds. Ever since, there has been a free emission of the gas. How long it will last, of course, cannot be determined; there seems to be no diminution in quantity since its first appearance.

The *burning* springs above referred to, have been bubbling up ever since the first white man set his foot upon this strange soil, without any diminution of energy. The amount expelled from them is no trifle—probably enough to furnish this city with lights. The gas which issues from them, as well as from the eight wells, is impure, though it burns with a beautiful flame. Before, as well as after, it is ignited, it exhales a most disagreeable sulphurous odour. J. P.

Louisville, February, 1844.

